# Noise risk assessments within the adequately controlled and reasonably practicable philosophies

**DOI:** 10.4102/hsag.v28i0.2457

**Published:** 2023-12-08

**Authors:** Oscar Rikhotso, Thabiso J. Morodi, Daniel M. Masekameni

**Affiliations:** 1 Department of Environmental Health, Faculty of Science, Tshwane University of Technology, Pretoria, South Africa; 2 Department of Occupational Health, Faculty of Health Sciences, University of the Witwatersrand, Johannesburg, South Africa

**Keywords:** administrative control, engineering control, cost-benefit analysis, precautionary principle, noise control, noise-induced hearing loss, occupational health and safety

## Abstract

**Background:**

The entire risk assessment process is fraught with methodological and technical uncertainties, exacerbated by the introduction in legislation of ambiguous technical terms such as adequately controlled and reasonably practicable. The combination of these factors renders the risk assessment process opaque regarding required employer actions for securing legal compliance within the noise risk assessment context.

**Aim:**

This study aims to evaluate how companies are applying and interpreting the adequately controlled and reasonably practicable philosophies within the context of hearing conservation programmes (HCPs) and noise risk assessment processes.

**Setting:**

Four manufacturing and utilities companies.

**Methods:**

The four companies, selected through convenience sampling, submitted noise risk assessment records for evaluation through document analysis to determine the companies’ interpretation of the adequately controlled and reasonably practicable philosophies.

**Results:**

In the reviewed noise risk assessment records, the adequately controlled and reasonably practicable philosophies were poorly discerned. Specifically, the hierarchical approach for noise control outlined in the noise induced hearing loss regulations, for which the basis for adequately controlled philosophy ensues, remains misinterpreted by employers. Furthermore, cost-benefit analysis, which enables decision-making on the tolerability of risk within the reasonably practicable philosophy, was also omitted in the assessments.

**Conclusion:**

The adequately controlled and reasonably practicable philosophies were poorly applied and interpreted by the participating companies, to the detriment of tangible noise control.

**Contribution:**

This study provides insights on company application and interpretation of the adequately controlled and reasonably practicable philosophies, and HCPs, which contributes to inaction on noise control.

## Introduction

The management of occupational health hazards and risks including occupational noise, as an example, is mandatory worldwide. In countries like South Africa, however, relevant legislation currently omits the prescription of specific and compulsory risk management principles for managing these risks (Bluff & Johnstone 2005). The omission also extends to an absent overarching risk assessment regulation such as that prescribed in the United Kingdom’s ‘The Management of Health and Safety at Work Regulations 1999’ (United Kingdom 1999). These policy weaknesses are faced by employers in South Africa during risk management endeavours. Apart from the implicit directive that a noise risk assessment be conducted before harm occurs, the assessment itself is also a requirement for assisting in the legal determination of whether exposure is controlled using the reasonably practicable test (Peace 2017). The reasonably practicable test requires the quantification of risks derived through a risk assessment and is weighed against the costs of instituting measures to control the risk (Bluff & Johnstone 2005; Peace 2017; South Africa 2003). The control of risks within the reasonably practicable approach remains a primary goal of occupational health and safety (OHS) professionals including occupational hygienists (Lyon & Popov 2019). Ultimately, a risk assessment enables employers to demonstrate the reasonably practicableness of current controls in addition to the continuous monitoring of changes in risk levels inclusive of the workplace, work type and employees (Peace 2017). Practically, a noise risk assessment records exposure factors such as ‘the degree of harm that might be caused, current implemented and future exposure controls for risk elimination or minimisation and associated costs’ (Peace 2017; South Africa 2003). Once in possession of the risk assessment information, employers can address the question of ‘has this risk been minimised so far as reasonably practicable?’ through the aid of professional judgement, risk prioritisation scales derived from structured consequence and likelihood matrices. However, the use of professional judgement compared to structured matrices, which is fraught with subjectivity, may be considered to breach the intended meaning of the reasonably practicable approach (Peace 2017).

The control of occupational noise in South Africa ensues from the outcomes of a noise risk assessment, which requires employers to approach control outlined in Regulation 10 of the noise induced hearing loss regulations factoring the requirements of the adequately controlled and reasonably practicable philosophies (South Africa 2003). The reasonably practicable philosophy contains factors that are included as part of the risk assessment (Kotre 2022). However, it remains unclear whether the regulated industry has approached compliance with the prescripts of these philosophies in mind, in view of the dearth of scientific research regarding the topic. The function of enforcing and providing guidance on these philosophies is assigned to the Department of Labour and Employment Inspectorate in South Africa, which uses a combination of passive and reactive enforcement regimes (Hood et al. 1999). The prevailing social, economic and political climate, however, affects how enforcement is carried out regarding how inspectors interpret various concepts justifying regulatory intervention. This has an impact on the effectiveness of the enforced law (Hutter 1993). State regulation itself mirrors the risk perception of regulators, reflected in the adoption of either high levels of risk tolerance or extreme risk aversion (Hood et al. 1999). Overall, the methods in which OHS regulations are executed, from regulator, employer and employee perspectives, remain poor because of inadequate decisions made by these stakeholders (Tidwell 2000).

The recognition and control of risks from work apply to both self-employed persons and employers, a duty fulfilled during the risk assessment process (South Africa 1993, 2003; Straub 2018). The risk analysis of a noise risk assessment process, which includes hazard identification and corresponding exposure control, influences the success of occupational health programmes (Balderson 2016) including hearing conservation programmes (HCPs). Hearing conservation programmes include the implementation of elements such as elimination, substitution, engineering control, administrative control, audiometry and training programmes, and hearing protection devices (HPDs). The noise regulations from South Africa (2003) and the United Kingdom (United Kingdom 2005) however do not explicitly require employers to implement HCPs compared to the United States’ noise standard (Occupational Safety and Health Administration 1995). However, performance standards in both South Africa and the United Kingdom, expressed in the form of noise limits, inform employers on the actions required to control exposure (Bluff & Johnstone 2005) to levels considered adequately controlled (South Africa 2003). In South Africa, the South African National Standard (SANS) 10083 (2021) code of practice does however describe the elements of an HCP. For clarity on this matter, reference to the SANS 10083 code of practice is only mentioned in Regulations 7 and 8 of the Noise Induced Hearing Loss Regulations describing specific measurement procedures for noise monitoring and audiometric testing. Even with the implementation of recommended exposure control to conserve hearing, there always remain residual risks (Balderson 2016), as the design of occupational health programmes including HCPs and risk management approaches may not necessarily eliminate all risks (Ivensky 2016). In view of that, within the reasonably practicable approach, employers are relieved of instituting controls deemed unreasonable for lowering exposure (Kotre 2022). With the above-implied interpretation, risk assessments are thus endangered of becoming useless without tangible risk treatment ensuing from its findings (Lyon & Popov 2019); as employers, the ultimate authority on OHS issues may opt for an easy way out.

In determining that risks are adequately controlled within the reasonably practicable approach, comparisons between the outcomes of a risk assessment and the sacrifice (inclusive of money, time and trouble) are completed (Health and Safety Executive [HSE] 2001). The reasonably practicable philosophy is however ambiguously defined and gives rise to uncertainties as judgements are based on subjective evidence (Ferrie 2009). To aid employers in addressing this murky concept, Safe Work Australia (2010) published a generic interpretive guide clarifying the elements constituting reasonably practicable. Nonetheless, employers are still required to conduct an adequate risk assessment to secure compliance with both the general duty clause (Bluff & Johnstone 2005) and Regulation 7 in the Noise Induced Hearing Loss Regulations in South Africa (2003). Based on the outcome of the reasonably practicable test, a workplace ‘can be deemed safe when one has *reasonable knowledge* and judges the risks to be acceptable’ (Tidwell 2000). Following the reasonably practicable test, a decision on the tolerability of residual risk is formed. The factors that determine risk acceptability, subjective in nature, depend on the country, socioeconomic status, political dynamics, legal context and organisational culture (Tchiehe & Gauthier 2017). Tchiehe and Gauthier (2017) also posited that vulnerable employees tend to be tolerant of risks, an area that may require companies and regulators to spotlight through information dissemination. However, the state of OHS is influenced by the moral choices of all stakeholders within the concerned organisation (Tidwell 2000).

The high incidence of occupational diseases such as noise-induced hearing loss (NIHL) indicates operational failures in processes used for predicting risk including noise risk assessments. Sound risk management processes should be established to avoid these operational failures. An example of risk management process includes HCPs, specifically instituted to manage noise risks. On this, it is clear that OHS management system processes only manage organisational risk (Tusca 2018b). The decision-making capacity of companies for preventing and predicting risk is, however, influenced by an organisation’s culture and risk management processes (Tusca 2018a). However, the determination of the state of exposure control within the reasonably practicable approach is supported by cost-benefit analysis (Kotre 2022), key principle in defining the risk acceptability and identifying methods for the reduction of risks. Similar to some federal states in Australia, the Noise Induced Hearing Loss Regulations in South Africa (South Africa 2003) are vague in addressing issues such as the reference to a relationship between reasonably practicable and risk management (Bluff & Johnstone 2005). This study evaluated how four South African companies are applying and interpreting the adequately controlled and reasonably practicable philosophies within the context of HCPs and noise risk assessment processes.

## Research methods and design

### Study design and setting

This qualitative research design was conducted in 2021 and explored the status of the companies’ application and interpretation of the adequately controlled and reasonably practicable philosophies within the context of HCPs and noise risk assessments. The participating companies had operational footprints in the Gauteng, KwaZulu-Natal, Limpopo and Mpumalanga provinces of South Africa.

### Company identification, selection, inclusion and record submission

Companies considered for participation in the study were identified through a literature review study, procedures and results that are described by Rikhotso, Morodi and Masekameni (2022). The four companies included in the study were operating in the manufacturing and utilities sectors and had confirmed historic NIHL disclosures. Documents from a total of 21 plants from the four companies covering the period between 2018 and 2021 were electronically submitted to the primary investigator (O.R.) for evaluation consideration. Of the 21 reports, 11 were from Company 1, 6 were from Company 3, whilst Companies 2 and 3 submitted 2 each. The integrity and confidentiality of the submitted reports were maintained by storage in a password-secured folder.

### Adequately controlled and reasonably practicable evaluation

The criteria for determining the status of adequately controlled philosophy were interpreted directly, using legal guidance stated in Subregulation 6(4) and Subregulation 10(1) of the Noise Induced Hearing Loss Regulations (South Africa 2003). The factors for determining reasonably practicable were also interpreted directly from Subregulation 10(2) of the same regulation, with the expanded descriptors adapted from Peace (2017). Section 1 of the *Occupational Health and Safety Act* offers a South African context of the definition of the broad factors or variables constituting the reasonably practicable philosophy (South Africa 1993). The factors for the adequately controlled and reasonably practicable philosophies are shown in Table 1a and Table 1b and Table 2a and Table 2b, respectively. The variables for the adequately controlled and reasonably practicable philosophies were read directly from each submitted report in line with the READ approach (Dalglish, Khalid & McMahon 2020), because of the qualitative nature of the information (Kayesa & Shung-King 2021). The iterative steps of the READ approach were used to aid the evaluation and included (1) readying the materials, (2) extracting the data, (3) analysing the data and (4) distilling the findings. The qualitative data from the evaluation were input into Microsoft Word tables as shown in Table 1a, Table 1b, Table 2a and Table 2b.

**TABLE 1a:** Risk assessment and adequately controlled status assessment at Company 1 plants.

Evaluation criteria	Plant										
	A	B	C	D	E	F	G	H	I	J	K
**Adequately controlled condition**											
■ Noise < 85 dBA	✘	✘	✘	✘	✘	✘	✘	✘	✘	✘	✘
■ Noise > 85 dBA with action other than HPDs	✘	✘	✘	✘	✘	✘	✘	✘	✘	✘	✘
**Hierarchy of control**											
■ Elimination	✘	✘	✘	✘	✘	✘	✘	✘	✘	✘	✘
■ Substitution	✘	✘	✘	✘	✘	✘	✘	✘	✘	✘	✘
■ Engineering control	✘	✘	✘	✘	✘	✔	✘	✘	✔	✘	✔
■ Administrative controls	✔	✔	✔	✘	✔	✔	✔	✔	✔	✔	✔
■ Hearing protection devices	✔	✔	✔	✘	✔	✔	✔	✔	✔	✔	✔
**Adequately controlled assessment**	✘	✘	✘	✘	✘	✔	✘	✘	✔	✘	✔

*Source:* South Africa, 2003, *Noise-induced hearing loss regulations (GNR.307)*, Government Printer, Pretoria, viewed 21 October 2015, from https://www.gov.za/sites/default/files/gcis_document/201409/224990.pdf.; South African National Standard, 2021, ‘The measurement and assessment of occupational noise for hearing conservation purposes’, in *South African National Standard 10083*, pp. 5–52, Standards South Africa, Pretoria.

dBA, A-weighted decibel.

✘, No -(not assessed); ✔, Yes (assessed).

**TABLE 1b:** Risk assessment and adequately controlled status assessment at Companies 2, 3 and 4 plants.

Evaluation criteria	Company 2 (Plant)		Company 3 (Plant)						Company 4 (Plant)	
	A	B	A	B	C	D	E	F	A	B
**Adequately controlled condition**										
■ Noise < 85 dBA	✘	✘	✘	✘	✔	✘	✔	✘	✘	✘
■ Noise > 85 dBA with action other than HPDs	✔	✘	✘	✘	NA	✘	NA	✘	✘	✘
**Hierarchy of control**										
■ Elimination	✘	✘	✘	✘	✘	✘	✔	✘	U	U
■ Substitution	✘	✘	✘	✘	✘	✘	NA	✘	U	U
■ Engineering control	✔	✘	✘	✘	✔	✘	NA	✘	U	U
■ Administrative controls	✔	✔	✔	✔	✔	✔	✔	✔	U	U
■ Hearing protection devices	✔	✔	✔	✔	NA	✔	NA	✔	U	U
■ **Adequately controlled assessment**	✔	✘	✘	✘	✔	✘	✔	✘	U	U

*Source:* South Africa, 2003, *Noise-induced hearing loss regulations (GNR.307)*, Government Printer, Pretoria, viewed 21 October 2015, from https://www.gov.za/sites/default/files/gcis_document/201409/224990.pdf.; Peace, C., 2017, ‘The reasonably practicable test and work health and safety-related risk assessments’, *New Zealand Journal of Employment Relations* 42(2), 61–78.

✘, No (not assessed); ✔, Yes (assessed); NA, Not applicable; U, Unclear (not indicated in risk assessment records because of limitation of tool used).

**TABLE 2a:** Reasonably practicable evaluation at Company 1 plants.

Evaluation criteria	Plant										
	A	B	C	D	E	F	G	H	I	J	K
**Severity and scope of the hazard or risk**											
**Likelihood of the hazard (cause) or risk occurring**											
■ Current state of knowledge about likelihood of harm	✔	✔	✔	✔	✔	✔	✔	✔	✔	✔	✔
■ Chronic or acute likelihood	✘	✘	✔	✘	✔	✘	✔	✘	✔	✘	✘
■ Uncertainties about likelihood of harm	✘	✘	✘	✘	✘	✘	✘	✘	✘	✘	✘
**Degree of harm (the consequences) that might result from the hazard or risk**											
■ Nature and severity of the potential harm	✔	✔	✔	✘	✔	✘	✔	✔	✔	✘	✔
■ Knowledge about harm of the nature and severity	✔	✔	✔	✘	✔	✘	✔	✔	✔	✘	✔
■ Immediate or delayed harm	✘	✘	✔	✘	✔	✘	✔	✔	✔	✘	✘
■ Is the harm reversible	✘	✘	✘	✘	✔	✘	✔	✔	✔	✘	✘
■ Uncertainties about the magnitude of the harm	✘	✘	✘	✘	✘	✘	✘	✘	✘	✘	✘
■ Expected number and range of harms arising from the hazard	✘	✘	✘	✘	✘	✘	✔	✘	✔	✘	✘
■ Detectability of adverse effects	✔	✔	✔	✘	✔	✔	✔	✔	✔	✔	✔
**State of knowledge reasonably available concerning hazard or risk**											
■ Current state of knowledge about means available to eliminate or minimise risk	✘	✘	✘	✘	✘	✘	✘	✔	✔	✘	✔
■ Availability of guidance documents on the hazard and associated risks (freely available or of restricted access)	✘	✘	✘	✘	✘	✘	✘	✘	✔	✘	✘
■ Exposed population aware of it and the potential harm it might cause	✔	✘	✔	✘	✘	✔	✔	✔	✔	✘	✔
**Ways of eliminating or minimising the hazard or risk**											
■ Reasonable possibilities of discovering new means to eliminate or minimise risk	U	U	U	U	U	U	U	U	U	U	U
■ Can hazard or potential harm be eliminated	U	U	U	U	U	U	U	U	U	U	U
■ Substitution of current hazard with lesser hazard, so reducing the risk	U	U	U	U	U	U	U	U	U	U	U
■ Hazard isolation from people	U	U	U	U	U	U	U	U	U	U	U
■ People isolation from hazard	U	U	U	U	U	U	U	U	U	U	U
■ Use of engineering control to minimise the hazard	U	U	U	U	U	U	U	U	U	U	U
■ Administrative control implementation to minimise risk	U	U	U	U	U	U	U	U	U	U	U
■ Benefit of PPE for residual risk	U	U	U	U	U	U	U	U	U	U	U
**The availability and suitability of ways to eliminate or minimise risk or hazard**											
■ Current controls over risk	✔	✘	✔	✘	✔	✔	✔	✔	✔	✔	✔
■ Effectiveness of current controls	✘	✘	✘	✘	✘	✔	✘	✘	✔	✘	✘
■ Person managing controls	U	U	U	U	U	U	U	U	U	U	U
■ Employee confidence in the quality of management	U	U	U	U	U	U	U	U	U	U	U
**The cost, including whether the cost is grossly disproportionate to the risk**											
■ Relatively cheap expenditure or modifications significantly reduce risk	✘	✘	✘	✘	✘	✘	✘	✘	✘	✘	✘

*Source:* South Africa, 2003, *Noise-induced hearing loss regulations (GNR.307)*, Government Printer, Pretoria, viewed 21 October 2015, from https://www.gov.za/sites/default/files/gcis_document/201409/224990.pdf.; Peace, C., 2017, ‘The reasonably practicable test and work health and safety-related risk assessments’, *New Zealand Journal of Employment Relations* 42(2), 61–78.

✘, No (not assessed); ✔, Yes (assessed); U, Unclear (not indicated in risk assessment).

**TABLE 2b:** Reasonably practicable evaluation at company plans 2, 3 and 4.

Evaluation criteria	Company 2 (Plant)		Company 3 (Plant)						Company 4 (Plant)	
	A	B	A	B	C	D	E	F	A	B
**Severity and scope of the hazard or risk**										
**Likelihood of the hazard (cause) or risk occurring**										
■ Current state of knowledge about likelihood of harm	✔	✔	✔	✔	✔	✔	✔	✔	U	U
■ Latency of likelihood	✔	✔	✘	✘	✘	✘	✘	✘	U	U
■ Uncertainties about likelihood of harm	✘	✘	✘	✘	✘	✘	✘	✘	U	U
**Degree of harm (the consequences) that might result from the hazard or risk**										
■ Nature and severity of the potential harm	✔	✔	✘	✘	✘	✘	✘	✘	U	U
■ Knowledge about harm of the nature and severity	✔	✔	✘	✘	✘	✘	✘	✘	U	U
■ Latency of harm	✔	✔	✘	✘	✘	✘	✘	✘	U	U
■ Is the harm reversible	✔	✔	✘	✘	✘	✘	✘	✘	U	U
■ Uncertainties about the magnitude of the harm	✘	✘	✘	✘	✘	✘	✘	✘	U	U
■ Expected number and range of harms arising from the hazard	✔	✔	✘	✘	✘	✘	✘	✘	U	U
■ Detectability of adverse effects	✔	✔	✔	✔	✔	✔	✔	✔	U	U
**State of knowledge reasonably available concerning the risk or hazard**										
■ Current state of knowledge about means available to eliminate or minimise risk	✘	✘	✘	✘	✘	✘	✘	✘	U	U
■ Availability of guidance documents on the hazard and associated risks (freely available or of restricted-access)	✘	✘	✘	✘	✘	✘	✘	✘	U	U
■ Exposed population aware of it and potential harm it might cause	✔	✔	✘	✘	✘	✘	✘	✘	U	U
**Ways of eliminating or minimising the hazard or risk**										
■ Reasonable possibilities of discovering new means to eliminate or minimise risk	U	U	U	U	U	U	U	U	U	U
■ Can hazard or potential harm be eliminated	U	U	U	U	U	U	U	U	U	U
■ Substitution of current hazard with lesser hazard, so reducing the risk	U	U	U	U	U	U	U	U	U	U
■ Hazard isolation from people	U	U	U	U	U	U	U	U	U	U
■ People isolation from hazard	U	U	U	U	U	U	U	U	U	U
■ Use of engineering control to minimise the hazard	U	U	U	U	U	U	U	U	U	U
■ Administrative control implementation to minimise risk	U	U	U	U	U	U	U	U	U	U
■ Benefit of PPE for residual risk	U	U	U	U	U	U	U	U	U	U
**The availability and suitability of ways to eliminate or minimise risk**										
■ Current controls over risk	✔	✔	✔	✔	✔	✔	✔	✔	U	U
■ Effectiveness of current controls	✔	✔	✔	✔	✔	✔	✔	✔	U	U
■ Person managing controls	U	U	U	U	U	U	U	U	U	U
**The cost, including whether the cost is grossly disproportionate to the risk**										
■ Relatively cheap expenditure or modifications significantly reduce risk	✘	✘	✘	✘	✘	✘	✘	✘	✘	✘

*Source:* South Africa, 2003, *Noise-induced hearing loss regulations (GNR.307)*, Government Printer, Pretoria, viewed 21 October 2015, from https://www.gov.za/sites/default/files/gcis_document/201409/224990.pdf.; Peace, C., 2017, ‘The reasonably practicable test and work health and safety-related risk assessments’, *New Zealand Journal of Employment Relations* 42(2), 61–78.

✘, No (not assessed); ✔, Yes (assessed); U, Unclear (not indicated in risk assessment records because of limitation of tool used).

### Data quality

Credibility was achieved by directly transcribing the content of the reviewed records, which are legal documents by context. Dependability was ensured by the aligned evaluation criteria that were derived from relevant legal prescriptions. Confirmability was ensured as the reported results reflect the information contained in legal records written by company representatives without the influence of the researchers. Transferability was ensured by using direct text taken from the submitted records during the discussion of the results.

### Ethical considerations

The Tshwane University of Technology’s Faculty Committee on Research Ethics-Science granted ethical clearance (FCRE 2020/10/015 [FCPS 02] [SCI]) for the study. Non-disclosure agreements were also signed between the primary investigator (O.R.) and the participating companies as applicable.

## Results

### Adequately controlled evaluation

The outcomes of the noise risk assessment indicating that employees are exposed to noise at or above the noise rating confirm that exposure has not been prevented at either company, reason for the recurring assessments. This then compels employers to satisfy the requirements of the adequately controlled philosophy. In this regard, exposure can only be considered adequately controlled if the noise exposure is below the noise rating limit of 85 dBA. Furthermore, the identification of the cause of the noise emission should be investigated from wherein action should be taken, excluding the consideration of the use of HPDs. Tables 1a and Table 1b show the evaluation of the qualitative outcomes of the adequately controlled status within the noise risk assessment context. For Company 1, only three plants (Plants F, I and K) had noise exposure status in line with the adequately controlled criteria, whereas Companies 2 and 3 (Table 1b) had one and two facilities that met the adequately controlled criteria, respectively. The risk assessment procedures used by Company 4 omitted recording of the variables used for determining the adequately controlled status of current exposure. Elimination, substitution and engineering controls were totally omitted as a control option at Companies 1, 2 and 4, whereas administrative control and HPDs were the default controls across all companies. In general, all the enrolled companies omit following the hierarchy of control steps, a pathway for determining whether the current exposure is adequately controlled.

### Reasonably practicable evaluation

Table 2a and Table 2b show the outcomes of the qualitative evaluation from the enrolled noise risk assessments against the reasonably practicable criteria. The knowledge about the current state regarding the likelihood of harm from exposure to noise, as well as the knowledge and means of detecting harm, is well documented at Companies 1, 2 and 3, with Company 4 risk assessment procedure not catering for the recording of this information. Overall, Company 4 adopted risk assessment methodologies omitted recording all information prescribed in Regulation 6 of the Noise Induced Hearing Loss Regulations (South Africa 2003), which is required for determining the reasonable practicableness of the control institution. The evaluation of the effectiveness of current controls, an important factor in risk analysis of the risk assessment process, was a common practice at Companies 2 and 4, whereas Company 1 risk assessment procedures omitted the evaluation of this variable. Furthermore, nearly all the noise risk assessments omitted recording the sources of noise exposure, another important factor in determining or identifying the means of removing or mitigating the hazard or risk. For all other variables constituting the reasonably practicable philosophy, the qualitative evaluation of the severity and scope of the hazard or risk; the state of knowledge reasonably available concerning the hazard; and the suitability and availability of means to remove or mitigate the hazard or risk showed varied outcomes across Companies 1, 2 and 3. This variability attests to variances in the output associated with each adopted risk assessment methodology. None of the enrolled company noise risk assessments indicated or included cost analysis, a major variable when determining the reasonable practicability of controls, which assists employers in decision-making regarding the tolerability of residual risk.

## Discussion

### Adequately controlled and reasonably practicable

Table 1a and Table 1b indicate that exposure in most of the facilities included in this study is not adequately controlled, and companies have omitted conducting the reasonably practicable test. The omission of this risk analysis step has also resulted in the case of companies having to rely heavily on administrative and HPDs as default control within HCPs. The traditional hierarchy of control described in Regulation 10 of the Noise Induced Hearing Loss Regulations in South Africa may be in part also to blame for the industry’s ineffective HCPs, as it excludes a layer of control dealing with the management of workplace culture (Ivensky 2016), which has been incorporated in the contemporary modified hierarchy of control philosophies. The management of workplace culture, an administrative control, ‘instils a belief that all accidents are preventable’ thereby presenting an area of improvement within the hierarchy of control philosophy, posited Ivensky (2016). In addition to the observed bias towards the use of administrative controls and the readily available HPDs within HCPs, the implementation of the much-needed engineering controls appears to be delayed by company efforts that overly concentrate on simple, high-frequency or probability and observable accidents (Ivensky 2016). Some chosen risk management approaches, such as HCPs established from incomplete risk assessment information, may result in negative outcomes (Aven 2004), such as NIHL incidence in the case of noise risk management. Workplace OHS programmes reliant on work procedures, training and personal protective equipment as the main exposure controls remain insufficient as worker attention is distracted by other competing work responsibilities (Beamer, McCleery & Hayden 2016; Tusca 2018a). To achieve absolute protection using these lower-order controls rely on employees’ 100% reliability during any exposure event (Tusca 2018a). To advance the implementation of effective exposure controls, worker activism promoting employee rights to a healthy and safe workplace can help force employers to devise effective plans and programmes. This created employee expectation will translate into programmes having to do more regarding the provision to employees of required protection rather than relying on the issuing of PPE, as is the case with HCPs where HPDs are the dominant control (Binney 1972).

There undoubtedly remains an inherent difficulty in defining the concept of ‘acceptable risk’ (Fischhoff 1983). A workplace can, however, be adjudged as safe or unsafe based on the reasonably practicable approach (Le Roux 2011). The reasonably practicable philosophy requires employers to expend additional financial resources for risk reduction, disproportionate to a decrease in risk (Balderson 2016). There are certain right decisions even though costs-benefit analysis indicates otherwise where costs outweigh the benefits (Tidwell 2000). It is thus incumbent upon the regulated industry to initiate processes to determine whether reasonably practicable has been achieved. The knowledge of these processes and the acceptability of risk by employees and company management increase the chance of programme success (Balderson 2016).

Unlike the HSE in the United Kingdom (HSE 2001), the Department of Labour and Employment in South Africa has no publicly stated guidance document on how its inspectors are interpreting or judging the acceptability of implemented or proposed measures within the reasonably practicable philosophy during enforcement activities. Such guidance, the HSE (2001) argued, can result in inspectors making consistent and transparent decisions. Within the HSE (2001) guideline, risk initially assessed as high would require employers to prove that the control of risks is reasonably practicable. Risk acceptability itself is influenced by current societal events, implying that a risk classified as acceptable presently may become unacceptable following an accident because of transient risk perception (Pike, Khan & Amyotte 2020). Risk ranked as tolerable is only tolerable following the implementation of cost-effective measures. However, tolerable risk in itself is against publicly stated slogans of the zero-harm concept, which can only be achieved with the effective elimination of all risks. The adoption of the zero harm concept by companies is contrary to the foundations of the very concept as neither moderate nor low risks are acceptable. Undoubtedly, zero harm is in contradiction to the reasonably practicable approach and compromises health and safety in that residual risk is acceptable. To the contrary, zero harm implies no compromise on health and safety (Ivensky 2016). Risk assigned in the as low as reasonably practicable region (ALARP) within the HSE tolerability of risk framework requires an evaluation of possible and viable exposure controls coupled with a post-assessment of which controls can be implemented (Aven, Vinnem, & Røed 2006). Under current realities, all workplaces should achieve and maintain a state of reasonably practicable as an ultimate goal (Lyon & Popov 2019). The use of the various available risk management methodologies, which focus on measuring and controlling risk, alone will not achieve workplace expectations of zero occupational disease incidence. More effort should also be directed towards building internal company capabilities for predicting and preventing risk (Tusca 2018b). In the absence of an authoritative decision-making guide regarding the uncertainty of understanding and the expression of risk, there remain differences within risk and decision analysis as risk analysts employ different approaches that may combine a risk acceptance criterion and the interpretation of cost-benefit analysis (Aven 2004). A healthy and safe workplace regarding noise can be achieved within the prescripts of HCPs as an example, when there are clearly defined criteria of acceptable residual risk (Tusca 2018a). The reasonably practicable philosophy in any instance qualifies the use of the hierarchy of control, which should be integrated in a risk assessment (Bluff & Johnstone 2005).

### Controls in risk assessments

Addressing occupational health risks requires employers to actively pursue the implementation of corresponding controls (Ferrie 2009). In this study, the documented controls are aligned with the default implementation of HCPs prior to the consideration of the adequately controlled and reasonably practicable philosophies. Employers implement controls as a fulfilment of the duty of care towards each employee. Should the courts find that the employer omitted to implement available controls that could have practically reduced risk before an accident occurred would represent a breach in the duty of care law (Bluff & Johnstone 2005). The fulfilment of the duty of care law by employers also requires the consideration that employees are fallible and careless at times. Regardless, employers are required to still take actions for preventing employees from suffering harm of their creation (Bluff & Johnstone 2005). Although the risk assessment is conducted on hypothetical persons, the practical application of control measures proposed in the assessment should be adapted to exposed employees and their abilities (HSE 2001). The identification of current and future controls, poorly followed in some of the enrolled companies, ensues from the entire risk analysis process and is fulfilled by employers in their quest for attaining legal compliance. The control of high risk and/or low cost risk limits employers on using excessive costs, company size and financial status as a basis for rejecting to implement measures (Ferrie 2009; HSE 2001). Currently, risk management approaches have tended to focus disproportionally on risk ranking with prevention and control of risks being a secondary matter (Bluff & Johnstone 2005). In this study, there remain limited indications that elimination, substitution and engineering control would be pursued in recurring noise risk assessment reviews. Seemingly, HCP implementation has been misconstrued to represent full legal compliance with the Noise Induced Hearing Loss Regulations (South Africa 2003).

### Exposure control and professional ethics

The exposure controls recorded in the enrolled noise risk assessment indicate the long-term view of employers towards noise control. Partly, the omission of adequately controlled and reasonably practicable tests is viewed as a fundamental contributor for the adoption of the observed low-order controls in this study. Decision-making ensuing from cost-benefit analysis, excluded by all companies in this study, promotes the ethical approach to exposure control as it introduces some form of fairness, transparency and honesty on decisions (Wachter 2011). Without introducing cost-benefit analysis as part of risk assessment, OHS professionals risk being labelled as ‘designated felons’ where organisations have adopted the regulatory approach to risk management (Wachter 2011). Regulation has created and placed the moral obligation for creating healthy and safe workplaces. The inferred disregard of workplace health and safety by employers towards employees raises ethical questions. This also highlights the intertwined relationship between health and safety and ethics, an area neglected by scholars in part because of being relegated as a secondary issue by employers. Employees, the exposed population, are also faced with an ethical choice regarding exposure to occupational risks (Tidwell 2000). The ethical approach within the OHS field pushes the moral bounds or limits of OHS specialists with regard to efforts required for reducing risks, against the backdrop of loss control approach used by employers in some instances. Even with the clear benefits of using cost-benefit analysis, the final decision of the acceptability of risk lies with employers, a dilemma for OHS professionals. This is so as there may be differences of professional opinion between managers and OHS professionals as to the acceptability of risk. (Wachter 2011). Wachter (2011) also posited that managers are willing to accept adverse events because of their sporadic occurrence. Ethical activism and education are proposed to entrench the view that only OHS professionals, with the support of other professions, can determine the acceptability of risk. This can also have a positive outcome in the image and continued justification of the various OHS professions (Wachter 2011).

In view of the uncertainties regarding the definition of reasonably practicable and the methodological issues from risk assessments, the precautionary principle offers another approach to exposure control (Pike et al. 2020). The precautionary principle has relevance in instances where there exist poor consequence predictions, risk descriptors or estimates (Aven 2006; Pike et al. 2020). The precautionary principle within the scope and ambit of noise risk assessments relates to the moral acceptability of the harm resulting from exposure. Moral acceptability covers the threat to human health from noise exposure, the irreversible nature of the health impact and the imposition of harm absent adequate consideration of employee rights. From the precautionary principle perspective and in view of the NIHL incidence, it is an inescapable and uncomfortable reality that the reasonably practicable philosophy has not resulted in zero risk or harm (Pike et al. 2020). With the noted shortcomings in the reviewed noise risk assessments, there is a pressing need for improving competency in risk assessments (Bluff & Johnstone 2005) in general.

### Study limitations

The study was conducted at only 21 consenting plants of four companies and can be extended to other companies in other sectors. There is a possibility that other companies with no reported historic NIHL incidence disclosures in publicly available sustainability reports might have been excluded from the study. Nonetheless, the study findings provide a new perspective on the noise risk management practices of companies regulated through the Noise Induced Hearing Loss Regulations in South Africa.

## Conclusion

In this study, the assessment and evaluation of the adequately controlled and reasonably practicable philosophies were omitted during the noise risk assessment processes of the participating companies. This has led employers to be reliant on lower-order controls, creating uncertainties regarding the technical and legal definitions of these concepts. The determination of the adequacy of controls within the noise risk assessment process follows the evaluation of the adequately controlled as well as the reasonably practicableness of current implementing controls. To improve the situation, risk assessors such as occupational hygienists should be adequately trained on the technical and legal aspects of the noise risk assessment process. Furthermore, the Department of Employment and Labour Inspectorate can consider issuing guidance documents clarifying these matters from a local context.
